# Screening active components from *Rubus amabilis* for pancreatic β-cells protection

**DOI:** 10.1080/13880209.2020.1787467

**Published:** 2020-07-13

**Authors:** Min Sun, Tiantian Zhu, Jinzhi Tong, Rezeng Caidan, Kaijin Wang, Guiqing Kai, Wenna Zhang, Lei Ru, Jiumei Pengcuo, Li Tong

**Affiliations:** a Anhui Provincial Key Laboratory of R&D of Chinese Material Medica, School of Life Science, Anhui University, Hefei, P. R. China; b College of Pharmacy, Qinghai Nationalities University, Xining, P. R. China; c Qinghai Jiumei Tibetan Medicine Co., Ltd., Xining, P. R. China; d Traditional Chinese and Tibetan Medicine Research Centre, Medical College of Qinghai University, Xining, P. R. China

**Keywords:** Procyanidin, PI3K/Akt/FoxO1 signalling pathway, apoptosis, cell membrane chromatography

## Abstract

**Context:**

*Rubus* species (Rosaceae) have been used in folk medicine to treat diabetes due to their hypoglycaemic activity.

**Objective:**

To screen the active components that act as hypoglycaemic agents in *Rubus amabilis* Focke and the underlying mechanisms.

**Materials and methods:**

Aqueous stem extract of *R. amabilis* was incubated with MIN6 β-cells, PBS was used as the blank control. Then the cells were washed, cell membrane-bound components were dissociated and identified by UPLC/MS. Total procyanidins (PCs) in *R. amabilis* was enriched and the cytotoxicity and anti-proliferation on β-cell were evaluated by MTT assay. PCs at 25, 50, and 75 μg/mL was applied for 24 h to determine its effects on palmitate (PA)-induced apoptosis and GSIS. Western blotting was employed to detect the protein expression of PI3K/Akt/FoxO1 signalling. The antioxidant indices were also measured.

**Results:**

β-Cell membrane-bound components were identified as three procyanidin B dimers and a C trimer. PCs showed no significant cytotoxicity up to a concentrations of 100 μg/mL. PCs treatment reversed the elevated apoptosis rate and impaired GSIS induced by PA. PCs markedly decreased the intracellular ROS and MDA production and increased the SOD activity. Moreover, PCs promoted the phosphorylation of Akt and FoxO1, and regulated Pdx-1 and Bax expression in MIN6 cells.

**Discussion and conclusion:** The active components that act as hypoglycaemic agents in *R. amabilis* are procyanidins, which protected MIN6 cells against PA-induced apoptosis by activating PI3K/Akt/FoxO1 signalling. These results indicate that β-cell extraction, combined with UPLC/MS, is a valid method for screening antidiabetic components from herbal medicines.

## Introduction

*Rubus* (Rosaceae) is composed of more than 600 species worldwide and has been cultivated for centuries for their fruits. In addition, numerous species are used in the folk medicine of many countries as hypoglycaemic remedies to treat diabetes (Krauze-Baranowska et al. 2010). *Rubus amabilis* Focke, *Rubus niveus* Thunb., and *Rubus sachalinensis* Lévl. are three commonly used *Rubus* species in Tibetan medicine in China. Previous studies revealed that *Rubus* species contained a variety of compounds, such as anthocyanins, ellagic acids (Caidan et al. 2015), terpenoids (Zhang et al. 2016; Chen et al. 2017), polysaccharide (Diao et al. 2018), flavonoids (Ren and Bao 2016), and polyphenols (Patel et al. 2004). Both clinical and experimental studies have reported that *Rubus* species possess hypoglycaemic activities, which are related to elevated insulin secretion (Lemus et al. 1999; Cheang et al. 2016). In addition, pharmacological studies have revealed that ellagic acids (Caidan et al. 2015), flavonoids (Patel et al. 2004; Caidan et al. 2015), and polyphenols (Diao et al. 2018; Wajs-Bonikowska et al. 2017) exert antioxidant effects. However, the active components that act as hypoglycaemic agents in *Rubus* and the underlying mechanisms remain unclear.

Pancreatic β-cells, a type of endocrine cell that secretes insulin and regulates blood sugar, play a central role in the pathogenesis of diabetes. Both type 1 and type 2 diabetes mellitus (DM) are characterized by progressive β-cell failure (Kahn 2000). When β-cells are damaged by various factors, absolute (D1M) or relative (D2M) insufficient insulin secretion follows, ultimately resulting in hyperglycaemia and diabetes. Apoptosis is the main form of β-cell death in both types of the disease (Cnop et al. 2005). Accordingly, the therapeutic strategy designed to arrest apoptosis is the most fundamental principle for both prevention and treatment of diabetes, and this approach may reverse the disease to some extent rather than just palliate glycaemia (Butler et al. 2003). The results of our preliminary experiments showed that an aqueous extract of *R. amabilis* could inhibit islet β-cell apoptosis. Therefore, we hypothesized that the hypoglycaemic effect of *R. amabilis* is achieved by protecting β-cells from apoptosis.

Cell membrane chromatography (CMC), which detects the bound components from cell extractions via liquid chromatography, is a convenient, specific, and time‐saving technique for screening active components from complicated herbal medicines. Some potential active components in Chinese medicine have been screened using various cell extractions, including mesangial cell (Sun et al. 2015), hepatocyte (Hong et al. 2012), epithelial cell (Shen et al. 2014), and macrophage (Yu et al. 2007) extraction. In this paper, the potential active components in *R. amabilis* were screened using pancreatic β-cell (MIN6 cell line) extraction, and the protective effects of the screened components were further examined using palmitate-treated MIN6 cells.

## Materials and methods

### Materials and chemicals

Stems of *R. amabilis* were collected in Guoluo, Qinghai Province, China, in April 2015 and identified by Professor Xuefeng Lu, Northwest Institute of Plateau Biology, Chinese Academy of Sciences. A voucher specimen (M0150408) was deposited in the Anhui Provincial Key Laboratory for R&D of Chinese Material Medica, Hefei, China.

High glucose Dulbecco's modified Eagle’s medium (DMEM) was purchased from HyClone Laboratories, Inc. (Logan, UT, USA). Fetal bovine serum (FBS) was obtained from Lonza Science SRL (Montevideo, Uruguay). Acetonitrile, methanol, acetone and formic acid (HPLC grade) for UPLC were obtained from Sigma-Aldrich (St. Louis, MO, USA). Ultrapure water was purified using a Milli-Q Plus water purification system (Milford, MA, USA). Procyanidin B2 standard (purity > 98%) was purchased from Weikeqi Biological Technology Co., Ltd. (Chengdu, Sichuan, China), and 4-dimethylaminocinnamaldehyde (DMAC) was purchased from Macklin Biochemical Co., Ltd. (Shanghai, China). D-101 macroporous resin (Qingdao Haiyang Chemical Co.) was used for column chromatography (CC). Spots were detected by 5% ferric trichloride reagents, followed by heating. All other chemical reagents were of analytical grade unless otherwise noted.

Thiazolyl blue tetrazolium bromide (MTT), 2′,7′-dichlorofluorescin diacetate (DCFH-DA), Hoechst 33342, β-mercaptoethanol, bovine serum albumin (BSA) and sodium palmitate were obtained from Sigma (St. Louis, MO, USA). Polyclonal antibodies against p27, Akt, and Akt (Phospho-Ser473) were purchased from Signalway antibody company (College Park, MD, USA), polyclonal antibodies against FoxO1 and p-FoxO1 (phospho Ser256) were purchased from ImmunoWay Biotechnology (Plano, TX, USA), and polyclonal antibodies against Bax and Pdx-1 were purchased from Proteintech (Wuhan, Hubei, China). Mouse monoclonal β-actin antibody and all of the secondary antibodies used for Western blotting were obtained from Abmart (Hangzhou, Zhejiang, China). An electrochemiluminescence (ECL) kit and BCA protein assay kit were purchased from Thermo Scientific Pierce (Rockford, IL, USA). Superoxide dismutase (SOD) and malondialdehyde (MDA) kits were obtained from Jiancheng Bioengineering Institute (Nanjing, Jiangsu, China). An Annexin V-FITC-PI apoptosis detection kit and insulin ELISA kits were purchased from Absin Bioscience Inc. (Shanghai, China) and Mercodia (Uppsala, Sweden), respectively.

### Preparation of *R. amabilis* extracts for CMC

The crude herb was dried, powdered and sieved through a 40-mesh sieve. Powder (20 g) was soaked in distilled water at room temperature for 2 h and then extracted twice in an ultrasonic bath for 1 h at 80 °C. Finally, the combined filtrate was centrifuged at 3000 rpm for 10 min. The supernatant was collected and dissolved in the same volume of 2 × phosphate-buffered saline (PBS, pH 7.4). The final concentration of *R. amabilis* extract used for chromatogram analysis was 20 mg/mL. The fingerprint of *R. amabilis* extract was identified and quantified using ultra-performance liquid chromatography/time-of-flight mass spectrometry (UPLC/TOF-MS).

### Cell line and MIN6 cell binding assay

A mouse pancreatic β-cell line, MIN6, was obtained from American Type Culture Collection (ATCC, Rockville, MD, USA). Cells were seeded in flasks and cultured in high-glucose DMEM containing 15% FBS, 10 µL/L β-mercaptoethanol, 100 U/mL penicillin and 100 μg/mL streptomycin in a humidified atmosphere of 5% CO_2_ at 37 °C. When cells were grown to 90% confluence, they were starved with a serum-free medium for 2 h. The spent medium was discarded, and cell binding was conducted via incubation with *R. amabilis* extract (20 mg/mL in PBS) for 45 min at 37 °C, followed by washes with PBS (pH 7.4). The washing step was terminated when no detectable compound was found in the fingerprint of the washing eluate. Finally, the remaining MIN6 cells were dissociated in 5 mL of PBS (pH 3.9, adjusted by phosphoric acid) at 37 °C for 30 min. All sample analyses were performed three times.

A blank sample of dissociated eluate, in which *R. amabilis* extract was replaced by PBS (pH 7.4), was generated using the method described above.

### Solid-phase extraction

Solid-phase extraction (SPE) was used to concentrate and enrich the binding compounds before analysis. The sample was transferred into a pre-equilibrated SPE column cartridge (250 mg, 6 mL). After washing the loaded cartridge with 1 mL of ultrapure water, the analysts were eluted with 500 µL of methanol and filtered by a 0.45 μm nylon membrane filter before UPLC/TOF-MS analysis. The final washing eluate, blank dissociated eluate, and dissociated eluate of MIN6 cells were concentrated by SPE columns. The C18 SPE extraction columns were purchased from Hanbon Science and Technology Co., Ltd. (Huaian, Jiangsu, China).

### UPLC/TOF-MS analyses

A Waters Acquity UPLC System (Milford, MA, USA) equipped with a vacuum degasser, a quaternary gradient pump, an autosampler, a column temperature controller, a diode-array detector (DAD) and an electrospray ionisation (ESI) ion source was employed in the experiments. The system was controlled by MassLynx software version 4.0. Chromatographic separations were performed on a C18 column (Zorbax, Eclipse plus C18, 2.1 × 100 mm, 1.8 μm, Agilent, Santa Clara, CA, USA). The mobile phase contained 0.5% formic acid-water (A; 100: 0.5, *v/v*) and methanol-acetonitrile (B; 1:1, *v/v*), and was pumped at a flow rate of 0.3 mL/min. The gradient was as follows: 0–1.5 min, 10–15% B; 1.5–12.0 min, 15–25% B; 12.0–17.0 min, 25–10% B. The column temperature was maintained at 30 °C. The detection wavelength was set at 254 nm and the injection volume was 10 µL.

UPLC coupled to a Waters Micromass LCT Premier XE TOF-MS was used to identify bound compounds in the dissociated eluate. The mass spectrometry conditions were as follows: Ionisation mode, ESI positive; capillary voltage, 2500 kV; cone voltage, 100 V; desolvation temperature, 350 °C; source temperature, 110 °C; desolvation gas flow, 600 L/h; cone gas flow, 50 L/h. The scan range was 80–1000 *m/z*.

### Extraction of procyanidins from *R. amabilis*

The dried stems of *R. amabilis* were ground to approximately 40 mesh by a disintegrator and 1.0 kg of the powder was extracted with 50% acetone (7 L × 3) at room temperature overnight. Then the extract solution was evaporated *in vacuo* to obtain an extract. The diluted extract was subjected to a D-101 macroporous resin column (Ф 1.6 × 60 cm, Huxi Analytical Instrument Factory Co. Shanghai, China) and eluted with water, 20% ethanol, and 40% ethanol. The content of procyanidins (PCs) was detected using the DMAC method, and the 20% ethanol fraction was combined and evaporated to dryness at 50 °C *in vacuo* and stored in a refrigerator. The total procyanidins content in the samples was >95%.

### Determination of PCs in *R. amabilis* using DMAC

The PCs content was measured using the method of Prior et al. (2010). DMAC assay was performed in 96-well plates. Briefly, the fractions of effluent from the D-101 macroporous resin column were collected and diluted. Each well contained 70 µL of sample and 210 µL of DMAC solution (0.1 wt% in acidic ethanol). Then, 91% ethanol (70 µL) was used as a blank, and a standard curve was produced using procyanidin B2 in 91% ethanol. The 96-well plate was shaken gently at 25 °C for 30 min, and the absorbance was then read at 640 nm using a Synergy HI Multi-Mode Microplate Reader (BioTek Instruments, Inc., Winooski, VT, USA).

### Cell treatments

Cells were cultured as described above, and the apoptosis of MIN6 cells was induced by culturing cells with a 0.5 mM palmitate/BSA complex (PA) for 24 h. PA was prepared as described in the literature (Hao et al. 2015). Sodium palmitate was dissolved in PBS at 70 °C for 30 min to obtain a 10 mM stock solution, and 5 mL of 20% (*w/v*) BSA solution was then added dropwise to 5 mL of sodium palmitate stock solution at 55 °C in a shaking water bath, followed by a 10 min incubation at 55 °C. The PA solution was cooled to room temperature and diluted 1:10 to a final concentration of 0.5 mM.

### Cytotoxicity and proliferation assay

The cytotoxicity and proliferation of MIN6 cells were determined by the MTT assay. MIN6 cells were plated into 96-well plates at a density of 5000 cells/well and cultured for 24 h. To detect the cytotoxicity of PCs extracted from *R. amabilis*, the cells were subsequently incubated with 12.5–100 μg/mL PCs for 48 h. For the proliferation assay, cells were subsequently treated with 0.5 mM PA in the presence or absence of PCs at different concentrations for 24 h. Then, 20 µL of MTT (5 mg/mL) was added to each well. After a 4 h incubation period, the media was removed and replaced with 150 µL of DMSO, and the plate was then shaken for 15 min at room temperature to ensure that all crystals had dissolved. The optical density (OD) was measured at 490 nm using a Synergy HI Multi-Mode Microplate Reader. The cell proliferation or cytotoxicity was expressed as the percentage of the control cells.

### Assessment of apoptosis

#### Flow cytometry

After 24 h of treatment, MIN6 cells were trypsinized and washed with PBS. Apoptosis was assessed using a commercial Annexin V-FITC-PI apoptosis detection kit (Absin, Shanghai, China). The procedures were performed according to the manufacturer's recommended protocol for flow cytometry analysis. The percentage of apoptotic cells was evaluated by flow cytometry.

#### Hoechst staining of apoptotic nuclei

MIN6 cells were plated in glass-bottom dishes and treated with PCs for the indicated times. The culture media was removed, and the cells were washed with cold PBS three times. Then, the cells were fixed in 4% paraformaldehyde for 30 min, followed by permeabilization of cells with 0.1% Triton X-100 for 15 min. Cells were stained with Hoechst 33342 (5 mg/mL) for 15 min, and the nuclei were observed under a Leica DMi8 confocal microscope.

### Glucose-stimulated insulin secretion (GSIS) assay

The cells were seeded into 24-well plates (10, 000 cells per well) and incubated with 0.5 mM PA in the presence or absence of different doses of PCs for 24 h. Then the cells were washed and preincubated in Krebs buffer (KRB: 135 mM NaCl, 3.6 mM KCl, 0.5 mM NaH_2_PO_4_, 0.5 mM MgSO_4_, 1.5 mM CaCl_2_, 2 mM NaHCO_3_, 10 mM HEPES, and 0.1% *w/v* BSA, pH 7.4) for 1 h at 37 °C. Then, the cells were incubated in KRB containing 5 mM glucose and, subsequently, 25 mM glucose for 1 h at 37 °C. The insulin level in the incubation media was measured using an insulin ELISA kit (Mercodia, Uppsala, Sweden) according to the manufacturer’s instructions. Cells in the plate were lysed by RIPA buffer, and the protein concentration of the lysates was detected using a BCA assay kit. The insulin secretion was normalized by cellular protein content.

### ROS generation

Intracellular ROS generation was determined using the fluorescent probe DCFH-DA as described previously (Sun et al. 2015). MIN6 cells were seeded in a 96-well plate and treated with 0.5 mM PA in the presence or absence of PCs at different concentrations for 24 h. Then, the cells were exposed to DCFH-DA (10 μM) in the dark for 30 min at 37 °C. The fluorescence was read at Ex 485/Em 530 nm on a Synergy HI Multi-Mode Microplate Reader. Fluorescent levels were expressed as a percentage of the control.

### SOD activity and MDA contents

Cells were seeded in triplicate in a 24-well plate and treated with PCs as described above. After a 24 h treatment by PCs, the supernatants were collected and centrifuged at 3000 rpm for 5 min at 4 °C. SOD activities and MDA contents were measured using commercial assay kits according to the manufacturers’ instructions.

### Western blotting

Cells were cultured in dishes and treated with 0.5 mM PA in the presence or absence of PCs at different concentrations for 24 h. MIN6 cells were washed with ice-cold PBS and lysed on ice in RIPA buffer (50 mM Tris, pH 7.5, 150 mM NaCl, 2 mM EGTA, 2 mM Na_3_VO_4_, 1 mM PMSF) containing HALT protease/phosphatase inhibitor cocktail (Sangon Biotech, Shanghai, China). The cell lysates were centrifuged at 4 °C for 20 min at 14,000 rpm, and the supernatants were obtained to detect the protein concentration using the BCA protein assay. Equal amounts (30 μg) of protein were separated electrophoretically using 8–12% SDS-PAGE and transferred to nitrocellulose membranes. The membranes were soaked in blocking buffer for 1 h and then incubated overnight with primary antibodies. After washing with PBST, membranes were incubated in either goat anti-mouse IgG or goat anti-rabbit IgG conjugated to horseradish peroxidase, and immunoreactive bands were visualised using an enhanced ECL kit.

### Statistical analysis

Data are expressed as the means ± SEM and were analysed by GraphPad Prism 5.0 Software (San Diego, CA, USA). One-way analysis of variance (ANOVA) followed by the Student–Newman–Keuls test was used to determine the statistical significance. For all tests, *p* < 0.05 was considered statistically significant.

## Results

### Screening potentially active components in *R. amabilis* by MIN6 cell extraction combined with UPLS/TOF-MS

#### UPLC fingerprints of untreated and SPE and acid treated-*R. amabilis* extract

**Figure 1.**
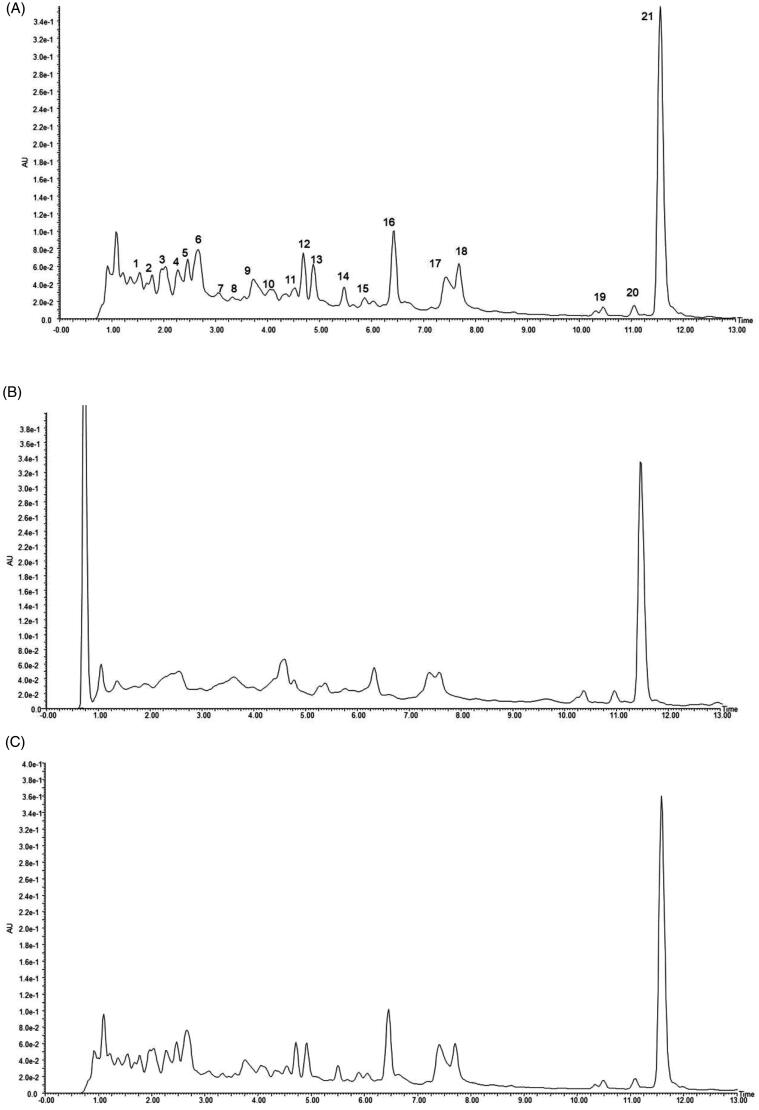
UPLC chromatograms of *R. amabilis* extract: untreated (A), after SPE (B), and after acid treatment (C).

To screen the active components in *R. amabilis* that interact with MIN6 cells, we first established a specific approach for the fingerprint analysis of *R. amabilis* based on UPLC detection. There were 21 main peaks in the fingerprint of the *R. amabilis* extract (Figure 1(A)). Typically, SPE is used to concentrate and enrich the dissolved eluate to reach the detection limit of the analytical instrument before analysis. Therefore, we subsequently compared the chromatograms of untreated and SPE-treated *R. amabilis* extract. After the SPE treatment (Figure 1(B)), both the heights and areas of the peaks were reduced in compared with those of the untreated samples (Figure 1(A)), illustrating the removal of several compounds by which the peak pattern is simplified.

We further studied the stability of *R. amabilis* extract during the incubation procedure. The pH value of the *R. amabilis* extract solution was adjusted to 3.9 by phosphoric acid, and the solution was incubated at 37 °C for 30 min to mimic cell dissociation conditions. The fingerprints of untreated and acid-treated *R. amabilis* extract were compared in Figure 1(A,C), and no obvious changes in the peak area or peak height were found in the acid-treated sample, indicating no obvious degradation under these conditions.

#### Analysis of binding components in MIN6 cell extraction

**Figure 2.**
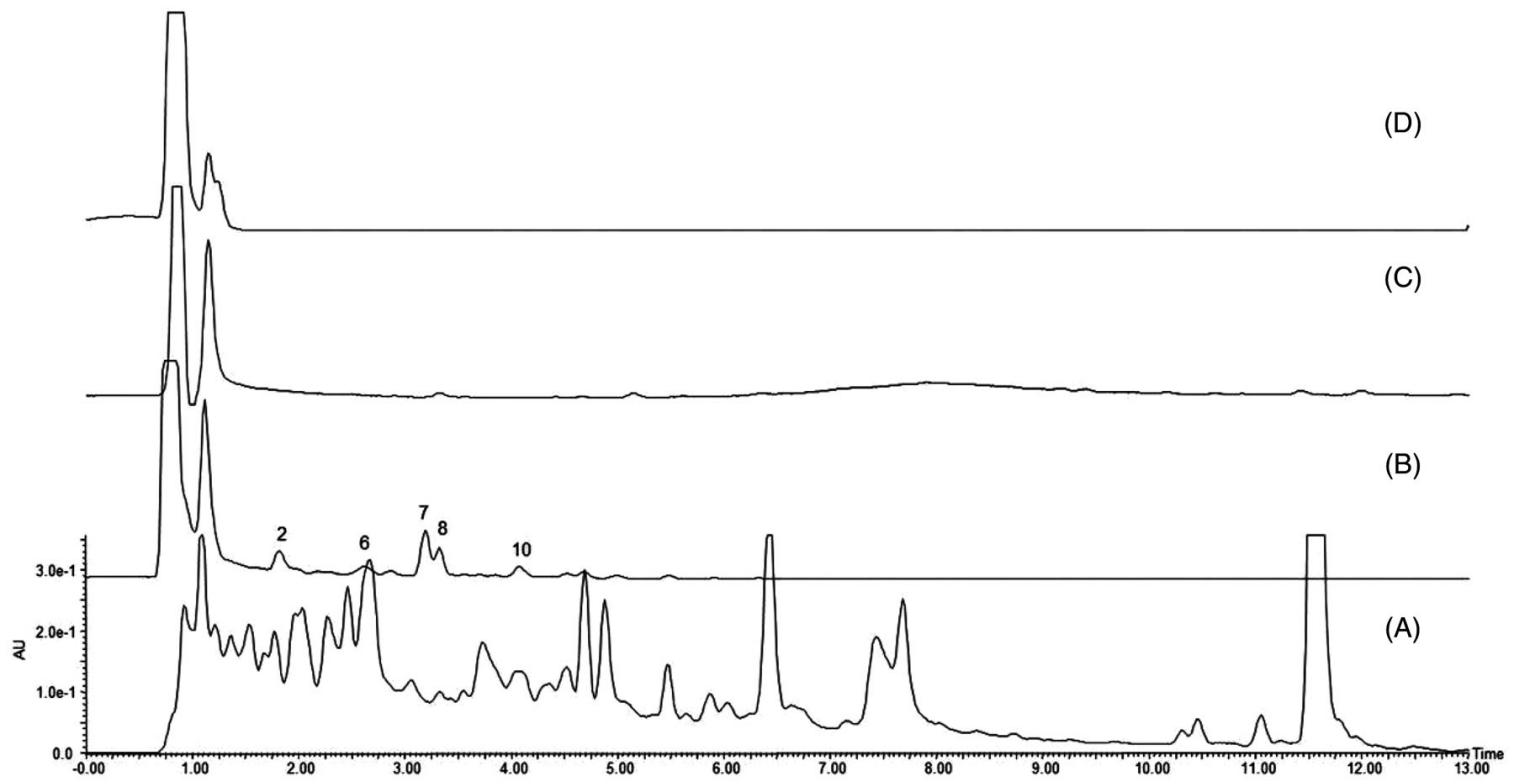
Characteristic chromatograms: (A) *R. amabilis* extract, (B) dissociated eluate of MIN6 cells, (C) final wash eluate of MIN6 cells, and (D) dissociated eluate of MIN6 cells incubated with PBS (blank).

Five main peaks were captured in the MIN6 cell-binding dissociated eluate (Figure 2(B)), and no specific peaks were found in the chromatograms of the final washing eluate and blank dissociated eluate (Figure 2(C,D)). All of the captured peaks in the dissociated eluate were determined by comparison with the chromatogram of *R. amabilis* (Figure 2). According to the theory of CMC, these five components are most likely to be the active compounds that bind to MIN6 cells.

#### Identification of binding components by UPLC/TOF-MS analysis

**Figure 3.**
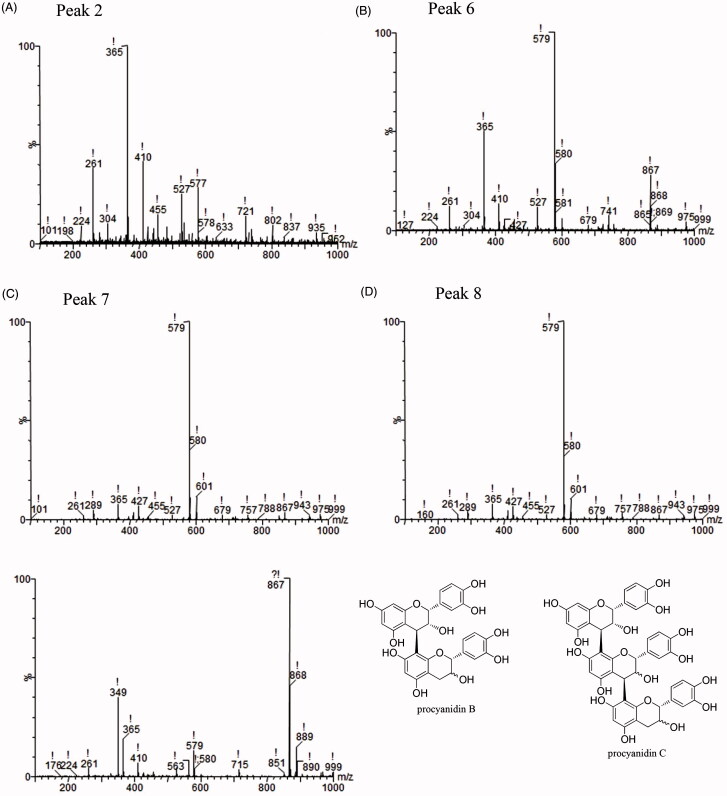
Identification of components in the MIN6 cell extraction by UPLC/TOF-MS. (A) ESI positive ionization spectrum of compound I. [M + H] ^+^ at *m/z* 365 is unknown. (B, C, D) ESI positive ionization spectra of compound 6(B), 7(C), 8(D) and their structural formulas (F). In positive ion mode, the product ion [M + H] ^+^ at *m/z* 579 is procyanidin B. (E) ESI positive ionization spectra of compound 10 and its structural formulas (F). In positive ion mode, the product ion [M + H] ^+^ at *m/z* 867 is procyanidin C.

UPLC/TOF-MS was applied to identify the above compounds. In positive ion mode, the ions [M + H]^+^ at *m/z* 579 were procyanidin B dimers (Figure 3(B–D)), and the ion [M + H]^+^ at *m/z* 867 was a procyanidin C trimer (Figure 3(E)). The ion [M + H]^+^ at *m/z* 365 was unidentified (Figure 3(A); Table 1).

**Table 1.** Identification of the components from dissociated eluate.

Peak no.	Retention time	Compound	Molecular formula	[M + H]^+^/ (*m/z*)
2	1.75	Unknown		365
6	2.67	Procyanidin B	C_30_H_26_O_12_	579
7	3.050	Procyanidin B	C_30_H_26_O_12_	579
8	3.317	Procyanidin B	C_30_H_26_O_12_	579
10	4.067	Procyanidin C	C_45_H_38_O_18_	867

### PCs from *R. amabilis* attenuated PA-induced apoptosis in MIN6 cells

#### Cytotoxicity and cell proliferation in MIN6 cell exposed to PCs

**Figure 4.**
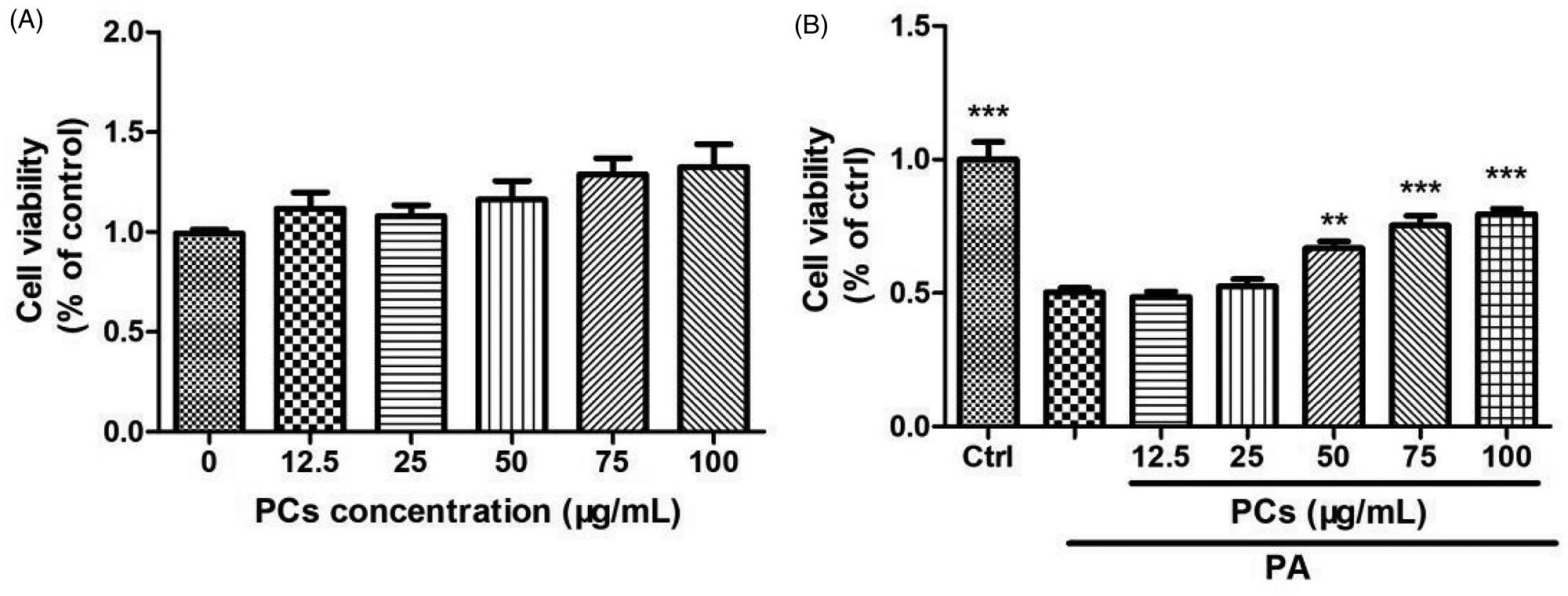
Cytotoxicity and cell proliferation in MIN6 cells exposed to PCs from *R. amabilis*. (A) To detect the cytotoxicity of PCs, MIN6 cells were incubated with 12.5–100 μg/mL PCs for 48 h. (B) For the proliferation assay, MIN6 cells were treated with 0.5 mM PA in the presence or absence of PCs at different concentrations for 24 h. An MTT assay was used to evaluate cell viability. The cell cytotoxicity and proliferation are expressed as the percentage of the control cells. Data are presented as the means ± SEM (*n* = 5) *p < 0.05, **p < 0.01, ***p < 0.001 vs. control group (in Figure A); vs. PA group (in Figure B).

As shown in Figure 4(A), there was no cellular cytotoxicity of PCs (12.5–100 μg/mL) on MIN6 cells during the 48 h incubation period. To examine the potential protective effects of PCs on MIN6 cells in a lipotoxicity model, cells were cultured in medium containing 0.5 mM PA for 24 h in the presence or absence of PCs at doses from 12.5 to 100 μg/mL. As shown in Figure 4(B), cell viability was decreased by 50% after 24 h of PA incubation (*p* < 0.05, vs. Ctrl). PCs treatment increased the cell viability of MIN6 cells, especially at doses from 50-100 μg/mL. Therefore, doses of 25, 50, and 75 μg/mL were used for follow-up experiments.

#### PCs protected MIN6 cells against PA-induced dysfunction and apoptosis

**Figure 5.**
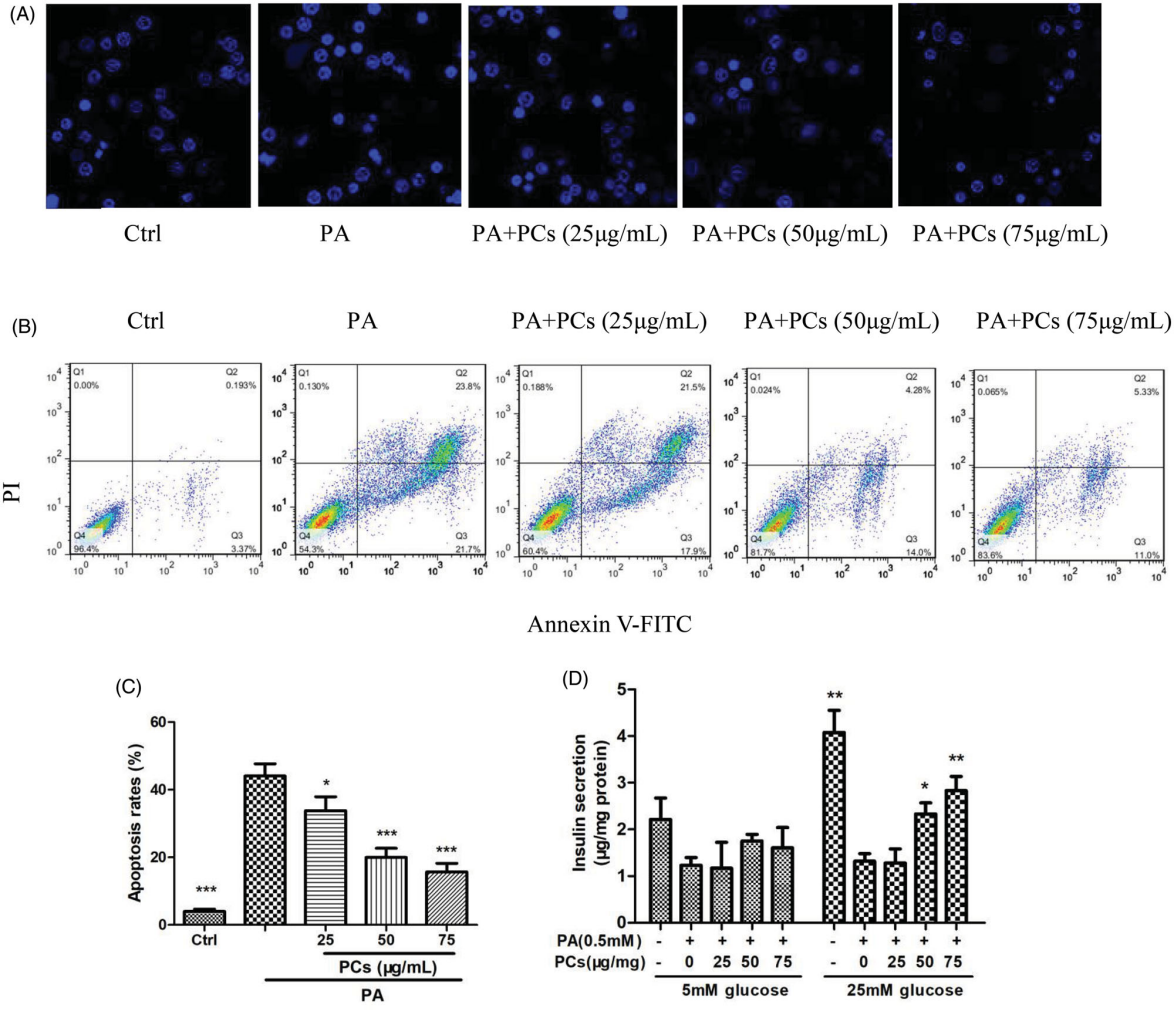
Effects of PCs from *R. amabilis* on PA-induced apoptosis in MIN6 cells. Cells were incubated with 0.5 mM PA in the presence or absence of PCs at different concentrations for 24 h. Then, (A) cells were stained with Hoechst 33342 and the nuclei were imaged. (B) The cells were double stained with Annexin V-FITC/PI, and the stained cells were then detected by flow cytometry. The fractions of cell populations different quadrants were analysed, and cells in the lower right quadrant (early apoptosis) and upper right quadrant (late apoptosis) represented apoptotic cells. (C) The percentages of total apoptotic cells in MIN6 cells. All experiments were performed in triplicate and expressed as the means ± SEM, *p < 0.05, **p < 0.01, ***p < 0.001 vs. PA group. (D) After MIN6 cells were incubated with PA in the presence or absence of PCs for 24 h, cells were stimulated with 25 mmol/L glucose. The supernatant was collected for an insulin assay. Means ± SEM, *n* = 3. *p < 0.05, **p < 0.01 vs. PA treatment only group.

To detect whether the PCs-induced promotion of cell proliferation increase in cell survival was attributable to apoptosis inhibition, we performed Annexin V-FITC/PI double staining and Hoechst 33342 nuclear staining at 24 h after treatment with PCs. As shown in Figure 5(A), PA incubation resulted in condensation and fragmentation, a specific morphological change associated with apoptosis. PCs treatment induced nuclear condensation and fragmentation. To quantify the degree of apoptosis in the various culture conditions, we performed double staining with Annexin V-FITC/PI. Apoptosis of MIN6 cells in response to PA combined with different concentrations of PCs was detected by flow cytometry (Figure 5(B)) and is summarised in Figure 5(C). As displayed in Figure 5(C), exposure of the cells to PA-induced apoptosis in a significant percentage of the cells, and apoptosis was significantly reduced in all PCs treatment groups compared with the 0.5 mM PA group (*p* < 0.05). Furthermore, apoptosis levels were significantly reduced in the 50 and 100 μg/mL PCs treatment groups compared with the 25 μg/mL PCs group (*p* < 0.05).

Moreover, PA incubation induced dramatically attenuated GSIS in response to 5 and 25 mM glucose. PCs treatment restored the impaired GSIS, most effectively at 75 μg/mL (Figure 5(D)).

#### PCs exerted antioxidant effects on PA-stimulated MIN6 cells

**Figure 6.**
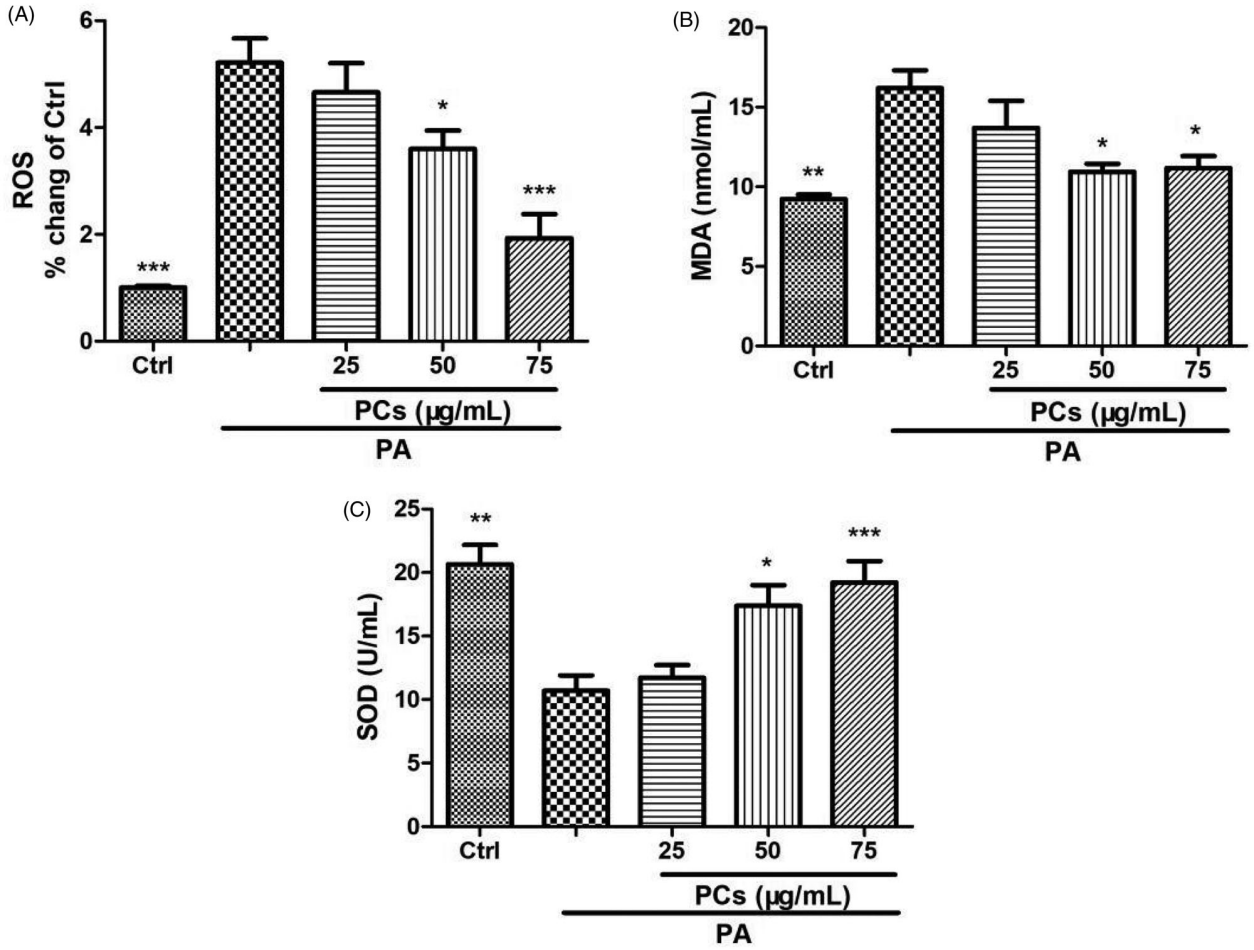
Antioxidant effect of PCs on PA stimulated MIN6 cells. Cells were exposed to PA in the presence or absence of PCs for the indicated times. (A) Intracellular ROS was measured by DCFH-DA. Culture media was collected for detecting, (B) MDA contents, and (C) SOD activity. Data are expressed as the means ± SEM (*n* = 3) of three independent experiments. *p < 0.05, **p < 0.01, ***p < 0.001, vs. PA group.

One important mechanism for PA-induced cell damage is oxidative stress. To investigate the effect of PCs on oxidative stress, PA-stimulated MIN6 cells were treated in the presence or absence of PCs (25–75 μg/mL) for 24 h. As depicted in Figure 6, PA incubation dramatically enhanced intracellular ROS and the production of MDA and suppressed the antioxidant status (SOD activity) in MIN6 cells. In contrast, treatment with PCs decreased the enhanced ROS and MDA levels and was associated with increased antioxidant levels.

#### PCs activated the Akt/FoxO1 signalling pathway

**Figure 7.**
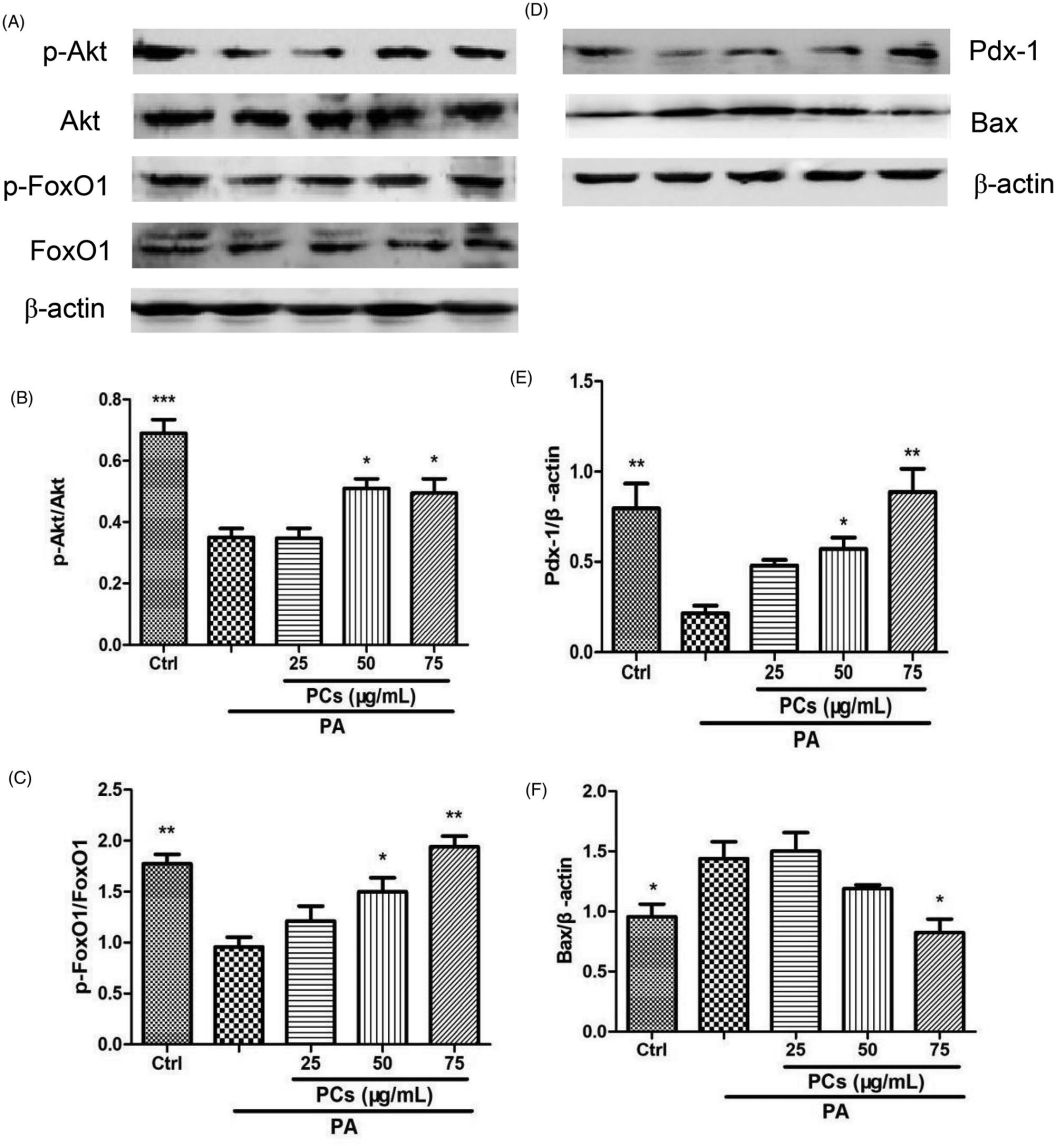
PCs induced activation of the Akt/FoxO1 signalling pathway. Cells were incubated with PA in the presence and absence of PCs for the indicated times. (A, D) Cell lysates from MIN6 cells were subjected to Western blotting analysis with the indicated antibodies. Densitometry analysis of (B) p-Akt/Akt, (C) p-FoxO1/FoxO1 expression, (E) Bax and (F) Pdx-1 expression. Levels of Pdx-1 and Bax proteins were normalised to β-actin. All of the experiments were repeated three times. The columns and error bars represent the mean and SEM (*n* = 3 per group). *p < 0.05, **p < 0.01, ***p < 0.001, vs. PA group.

The anti-apoptotic effect of PCs prompted us to investigate the underlying mechanisms. PA induces β-cell apoptosis via the phoshatidylinositol-3 kinase (PI3K)/protein kinase B (Akt)/Forkhead Transcription Factor (FoxO1) pathway. Thus, we next investigated whether the Akt/FoxO1 pathway is involved in PCs-mediated suppression of apoptosis. As shown in Figure 7(A,B), PA incubation resulted in a noticeable reduction in the phosphorylation of Akt (Ser473), and such a reduction was reversed by PCs at 50 and 75 μg/mL. FoxO1, one of the crucial downstream effectors of Akt, is required to regulate both replication and the response to oxidative stress in β-cells (Puddu et al. 2013). Subsequently, we tested the protein levels of phospho-FoxO1 (Ser256)/FoxO1. Compared with the control group, PA dramatically decreased the phosphorylation of FoxO1 (p-FoxO1/FoxO1). In contrast, PCs at 50 and 75 μg/mL treatment significantly increased the phosphorylation of FoxO1 (Figure 7(A,C)).

#### PCs regulated the expression of Pdx-1 and Bax

BCL2-associated X protein (Bax) and pancreatic duodenal homeobox-1 (Pdx-1) are target genes of FoxO1. Thus, we further examined Bax and Pdx-1 expression in MIN6 cells. PA remarkably upregulated the expression of Bax (Figure 7(D,E)) and downregulated the expression of Pdx-1 (Figure 7(D,F)). In contrast, these changes were reversed by PCs.

## Discussion

In the present study, we screened the active components in *R. amabilis* using MIN6 cell extraction and UPLC/TOF-MS and identified the active components as three procyanidin B dimers and a C trimer. Then, the PCs from the stems of *R. amabilis* was extracted. Finally, we tested the pharmacological effect of PCs on PA-induced MIN6 islet cell dysfunction and apoptosis. PCs from *R. amabilis* protected MIN6 cells against PA-induced dysfunction and apoptosis by increasing cell viability, suppressing oxidative stress, and activating the PI3K/Akt/FoxO1 signalling pathway.

CMC is a method for high-throughput screening of active components in herbal medicine (Hou et al. 2014). The active components are the pharmacodynamic basis of drugs. Herbal medicine exerts its effect by the interaction of its active compounds with membrane-bound targets, such as receptors, enzymes, and ion channels. Accordingly, cell membrane-bound compounds may be the potential active components in herbal medicine. The herbal medicine is usually taken after being boiled in water and used in folk medicine for their various properties, hence water decoction is used for cell binding by some researchers (Dong et al. 2005; Hong et al. 2012). This possibility can be examined further by analysing the dissociated eluate from the cell membrane using UPLC/HPLC-MS (Hou et al. 2014: Sun et al. 2015). According to the principle of CMC method, pancreatic β-cells and sulfonylurea receptors have been used to investigate the binding interactions between active ligands and their related targets (Ge et al. 2012; Zhang et al. 2013). The CMC method has been used to screen berberine from Cortex *Phellodendri* to target sulfonylureas receptors on β-cells (Bian et al. 2006). Mangiferin is also an active hypoglycaemic component and has a strong affinity for membrane proteins and abundant binding sites on pancreatic islet cell membranes (Shen et al. 2012). In the current study, β-cell membrane-bound active components were screened from *R. amabilis* and identified as three procyanidin B dimers and a trimer C, and procyanidins B were found to be highly abundant.

Procyanidins, the water-soluble and fat-soluble compounds, are the most abundant polyphenols found in foods such as fruits, red wine, green tea (Cádiz-Gurrea et al. 2014) and in bark and the seeds of many plants (Luo et al. 2018). Procyanidins arise from flavan-3-ol units, including catechin and epicatechin, and exist in a range of forms (dimers, trimers, tetramers, pentamers, and polymers). Based on the manner in which they are linked, the dimers are denoted B1 to B8, and the trimers are denoted C1 to C4. Among all types of procyanidins, the dimers are the most abundant in nature and have stronger antioxidant activities. Procyanidins also have anti-inflammatory (Youn et al. 2017), antioxidant (Luo et al. 2018), and hypoglycaemic properties (Gonzalez-Abuin et al. 2015). Therefore, they are used in the prevention and treatment of atherosclerosis, coronary heart diseases (Goszcz et al. 2017), DM (Gonzalez-Abuin et al. 2015; Ogura et al. 2016), and cancers (Choy et al. 2016). The hypoglycaemic effect of procyanidins is achieved by promoting peripheral glucose uptake, protecting islet β-cells and pancreas function (Pinent et al. 2004; Castell-Auví et al. 2012; Cedó et al. 2013), modulating the secretion of GLP-1 (Yamashita et al. 2013).

Persistent hyperglycaemia and hyperlipidaemia are the major factors associated with the loss of pancreatic β-cell mass and apoptosis, which are the core pathogenic mechanisms underlying D2M. Accordingly, saturated FFA such as palmitate is used to induce β-cell dysfunction and apoptosis to replicate the lipotoxicity model in D2M. Consistent with previous studies (Wang et al. 2014; Hao et al. 2015), our results showed that incubation with 0.5 mM PA for 24 h induced obvious apoptosis in MIN6 cells. PCs from *R. amabilis* significantly inhibited apoptosis, restored the impaired GSIS, and promoted β-cell proliferation, which implied that the PCs-induced promotion of cell proliferation and increase in cell survival were attributable to apoptosis inhibition. Similar findings have been reported by other research groups. Trimer procyanidins and cinnamtannin D-1, an A-type procyanidin oligomer isolated from cinnamon extracts, were verified to protect β-cells from PA and H_2_O_2_-induced dysfunction and apoptosis (Wang et al. 2014; Sun et al. 2016).

Furthermore, we studied the mechanism involved in the prevention of β-cell apoptosis by PCs. PA evokes the generation of ROS (Redza-Dutordoir and Averill-Bates 2016) and induces β-cell apoptosis via oxidative stress (Wang et al. 2014; Hao et al. 2015), which is represented by enhanced intracellular oxidant activity, an impaired antioxidant system and greater susceptibility to exogenous oxidative stress. ROS accumulation triggers apoptosis through PI3K/Akt/FoxO1 signalling pathways (Zhang et al. 2011; Hao et al. 2015). MDA is an endproduct of ROS-induced peroxidation. SOD, an antioxidant enzyme, can significantly scavenge free radicals, and its activity represents the intracellular antioxidation ability. High levels of ROS activate proapoptotic pathways, thus initiating programmed cell death. Our results showed that intracellular ROS levels and MDA concentrations were elevated upon exposure to PA, which could be suppressed by PCs treatment. In contrast, PCs increased PA-suppressed SOD activity.

The PI3K/Akt signalling pathway plays an important role in cell proliferation, apoptosis, and oncogenesis. Akt/PKB, an important downstream target kinase in the PI3K signalling pathway, binds to PIP3 and results in a conformational change, making Akt susceptible to phosphorylation at Ser473 and/or Thr308, which is required for Akt activation. Activated Akt further activates downstream factors, such as FoxO1. Suppression of the PI3K/Akt/FoxO1 signalling pathway has been implicated in PA-induced β-cell apoptosis (Shao et al. 2014; Hao et al. 2015). Therefore, we investigated whether the Akt/FoxO1 pathway is involved in PCs-mediated suppression of apoptosis. The results showed that PCs attenuated the PA-induced decreases in the levels of Ser473-phosphorylated Akt and Ser256-phosphorylated FoxO1, which suggested that PCs inhibited β-cell apoptosis, at least in part, via activation of the Akt/FoxO1 pathway. We further measured the expression of Pdx1 and Bax, downstream targets of FoxO1; the transcription factor Pdx1 is critical for maintaining the function of mature β-cells and is negatively regulated by FoxO1 (Cox and Kushner 2017), and the proapoptotic BCL-2 family member Bax is a critical regulator of the apoptotic pathway and is reportedly correlated with PA-induced β-cell apoptosis (Gurzov and Eizirik 2011; Shao et al. 2014). In this research, after incubation with PA, Bax was increased and Pdx-1was decreased, which could be reversed by PCs via the Akt/FoxO1 pathway, further confirming that PCs activated the PA-suppressed PI3K/Akt/FoxO1 signalling pathway.

## Conclusions

Taken together, the results of this study show that the procyanidin B dimers and C trimer are the active components from *R. amabilis*, as determined using the β-cell CMC method. PCs extracted from *R. amabilis* protects MIN6 cells against PA-induced dysfunction and apoptosis via their antioxidant capacity and activation of the PI3K/Akt/FoxO1 signalling pathway. These results provide a mechanistic basis for *Rubus* species as potential hypoglycaemic herbs to protect pancreatic β-cells, and demonstrate that β-cell extraction combined with UPLC/MS is a valid method for screening antidiabetic components from complicated herbal medicines.
